# Supplementary material for: AKT Signaling Modifies the Balance between Cell Proliferation and Migration in Neural Crest Cells from Patients Affected with Bosma Arhinia and Microphthalmia Syndrome
Source: Biomedicines. 2021 Jun 29;9(7):751. doi: 10.3390/biomedicines9070751 (PMC8301469; doi:10.3390/biomedicines9070751)
Supplement: Supplementary file 1 [file biomedicines-09-00751-s001.zip › Laberthonniere et al. Supplementary figures.pdf]

Supplementary figures

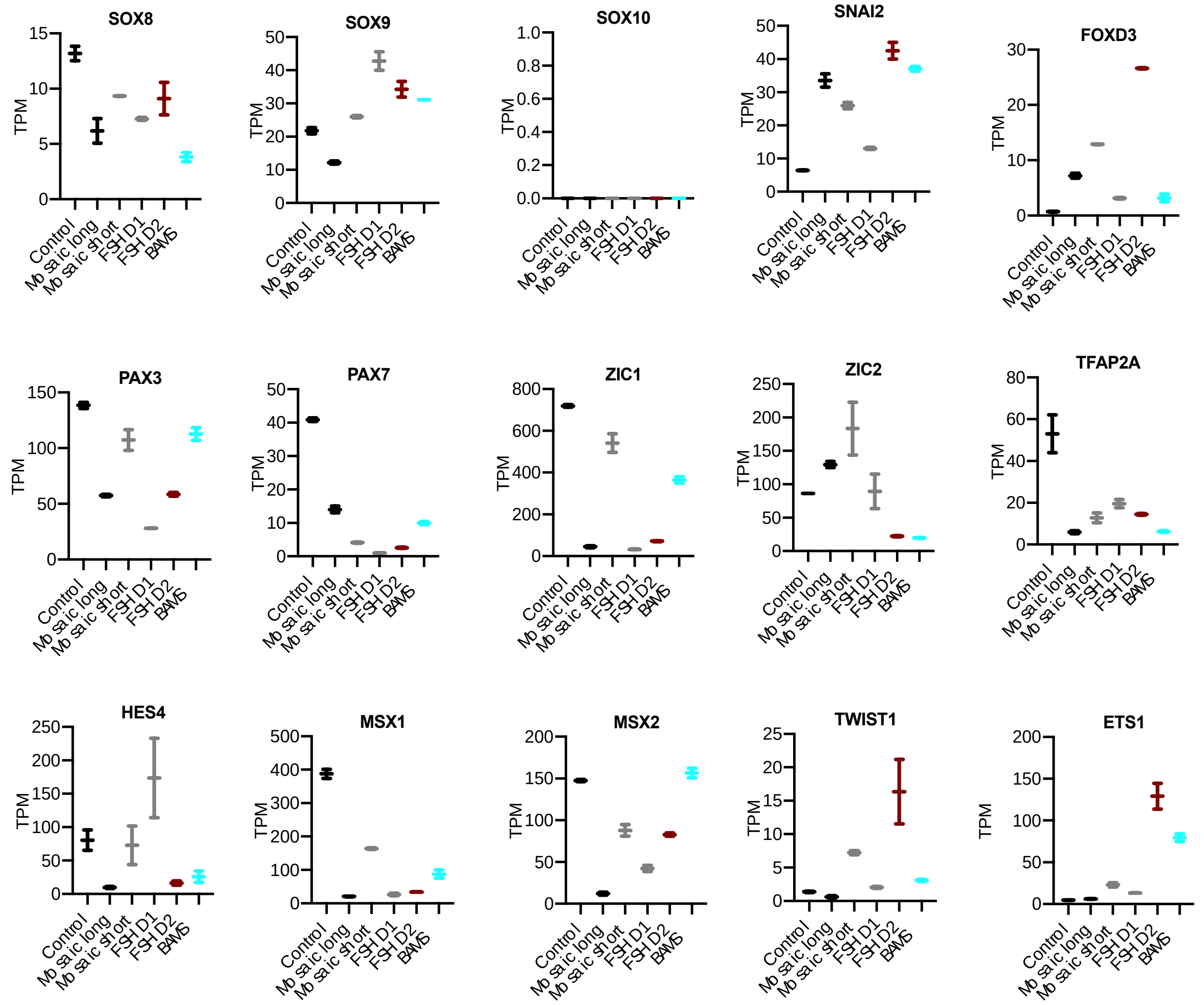

Supplementary figure S1. Expression of neural crest markers in hiPSC-derived NCSC. Transcript per Millions values for neural crest markers for each group of hiPSC-derived Neural Crest Stem Cells (NCSC) after RNA sequencing. FSHD1 short corresponds to a clone containing the contracted D4Z4 (3 RUs) from mosaic patient and FSHD1 long to its isogenic control (15 RUs).

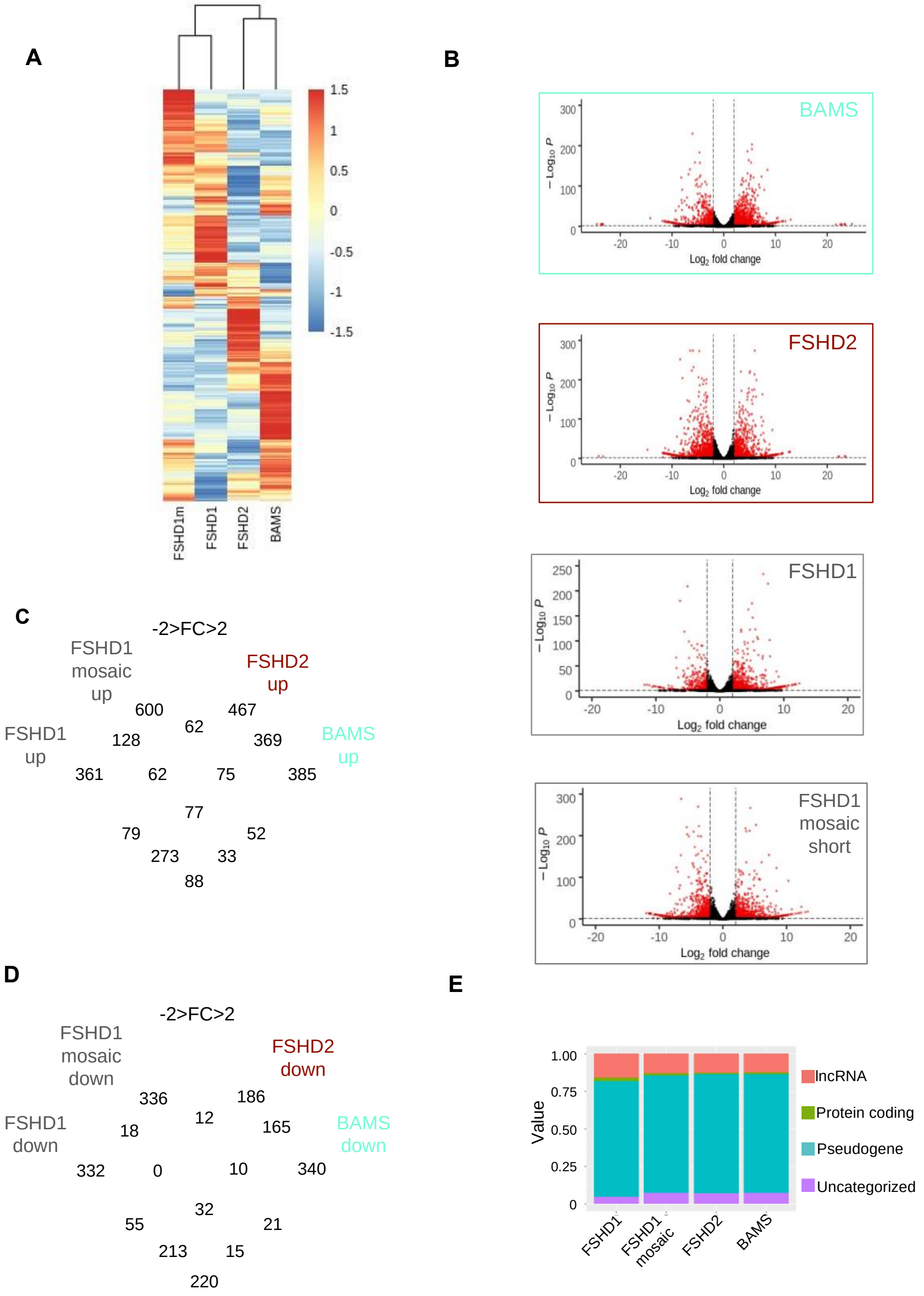

Supplementary figure S2. RNA Seq analysis reveals different pattern of expression in neural crest stem cells derived from BAMS or FSHD patients

A. Heatmap of RNAseq data (TPM values, distance: Manhattan, Clustering: Ward.D2) for gene expressed at D11 of differentiation in hiPSC-derived Neural Crest Stem Cells (NCSC). Unsupervised hierarchical clustering separate BAMS from FSHD patients (FSHD2, FSHD1 and FSHD1-mosaic). We did not find any sample with an adapter contamination > 0.1%. All samples had less than 1% of reads corresponding to overrepresented sequences. The % of bases above Q30 > 96.87 for all samples. Duplicated sequences were marked using Sambamba (v0.6.6) and excluded from final counting. **B.** Volcano plots for genes differentially regulated in BAMS, FSHD2 FSHD1 and FSHD1 mosaic clone versus controls. Fold changes (FC log 2) are compared to the number of reads (logCounts). Black dots represent genes that did not reach significance whereas dysregulated genes are shown in red. **C.** Venn diagrams for genes upregulated in hiPS-derived NCSCs from the different categories of patients compared to controls. **D**. Venn diagrams for genes downregulated in hiPS-derived NCSCs

from the different categories of patients compared to controls. **E**. Distribution of the different types of transcripts identified by RNA Seq. Pink, long non coding RNAs. Green, Protein coding RNAs. Cyan, pseudogenes. Purple, others.

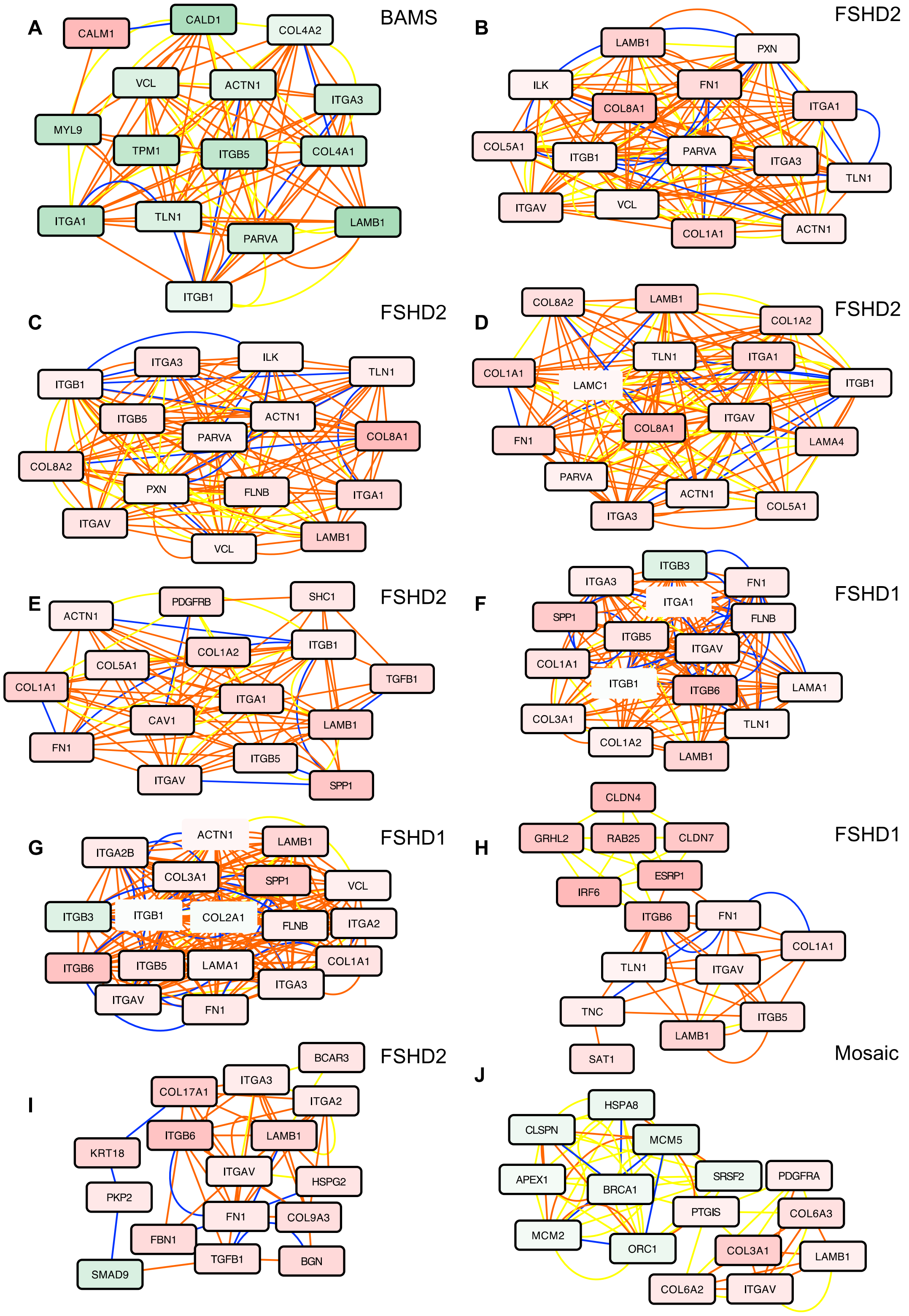

Supplementary figure S3. Active modules of genes encoding extracellular matrix (ECM) components and sarcomeric proteins involved in muscle contraction. Representative active modules sampled from the accumulated Pareto front of 30 runs for BAMS (**A**), FSHD2 (**B**-**E**), FSHD1 (**F**-**I**) or FSHD1 mosaïc (**J**) datasets using the MOGAMUN algorithm. The color of the edges (links) denotes the type of interaction/relationship between each pair of genes. Specifically, protein-protein interactions (blue links), biological pathways (orange links) and correlation of expression data (yellow links). Up-regulated nodes are colored

in red and down-regulated ones, in green, the intensity of the color reflects the fold-change. The black border reflects the level of significance (FDR<0.05 and -2>FC>2)**.** Each active module contains between 15 and 16 genes. Genes corresponding to each nodes were analyzed using g:Profiler to define corresponding molecular function and p-value. **A.** ECM organization in BAMS, p-value $2.12e^{-7}$. **B.** ECM organization in FSHD2, p-value $1.1e^{-17}$**. C.** ECM organization, FSHD2, p-value $2.36e^{-15}$. **D.** ECM organization, FSHD2, p-value $1.84e^{-16}$. **E.** ECM organization, FSHD2, p-value $7.2e^{-15}$. **F.** ECM organization, FSHD1, p-value $8.6e^{-19}$. **G.** ECM organization, FSHD1, p-value $1.6e^{-6}$. **H.** ECM organization, FSHD1, p-value $1.4e^{-19}$. **I.** ECM organization, FSHD1, p-value $2.8e^{-19}$. **J.** ECM organization, FSHD1 mosaic, p-value $7.06e^{-4}$.

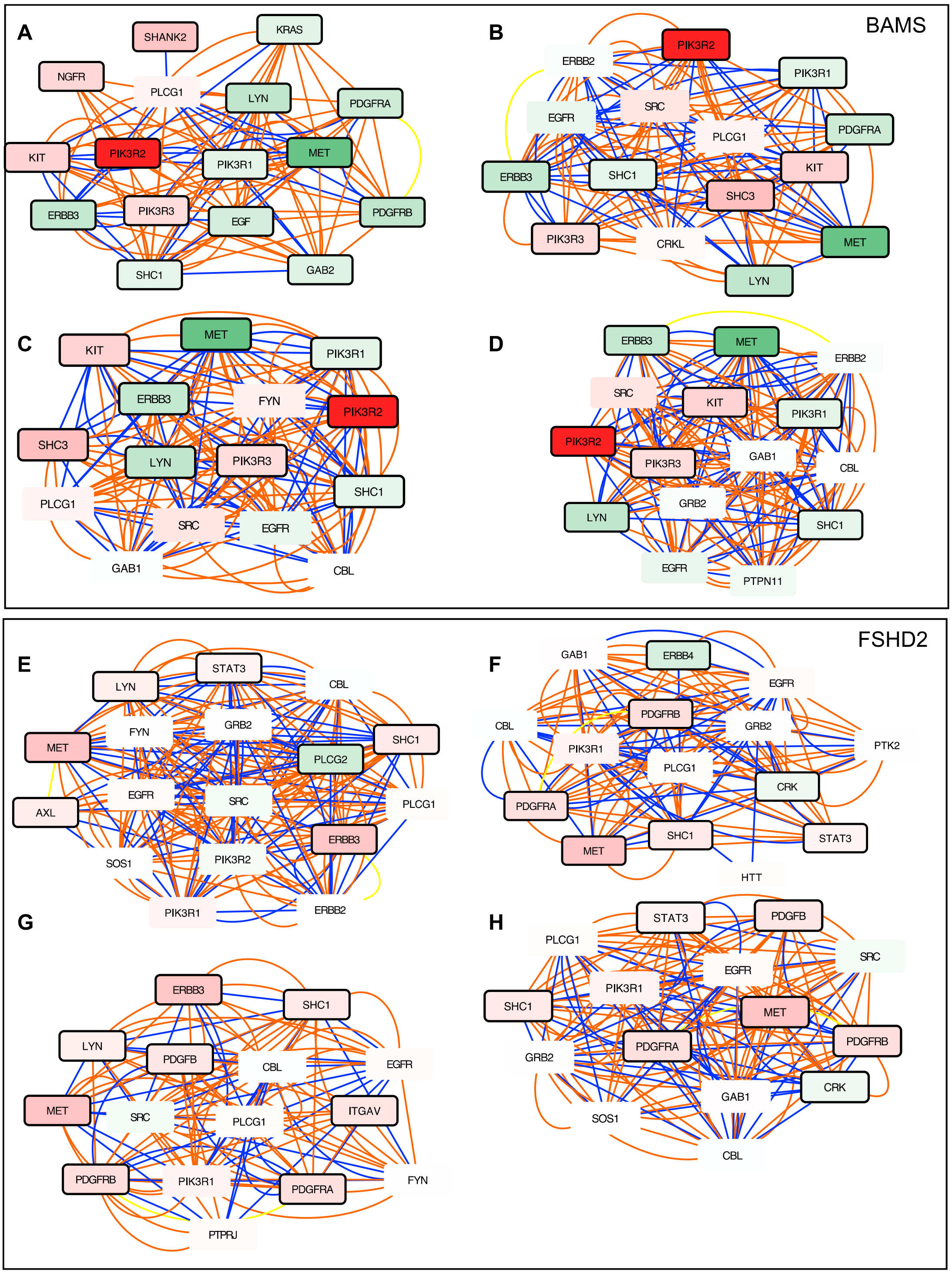

Supplementary figure S4. Active modules of genes involved in transmembrane receptor Tyrosine kinase signaling in BAMS and FSHD2 cells. Representative active modules sampled from the accumulated Pareto front of 30 runs for BAMS (A-D) and FSHD2 (E-H) datasets using the MOGAMUN algorithm. The color of the edges (links) denotes the type of interaction/relationship between each pair of genes. Specifically, protein-protein interactions (blue links), biological pathways (orange links) and correlation of expression data (yellow links). Up-regulated nodes are colored in red and down-regulated ones, in green, the intensity of the color reflects the fold-change. The black border reflects the level of significance (FDR<0.05 and -2>FC>2). **A-D.** BAMS cells. **A.** p-value $8.51e^{-19}$**. B.** $3.29e^{-18}$. **C.** $3.29e^{-18}$. **D.** $3.29e^{-18}$. **E-F.** FSHD2 cells, **E.** p-value $8.\ 95e^{-16}$**. F.** $1.141e^{-15}$**. G.** $3.29e^{-18}$. **H.** $3.29e^{-18}$.

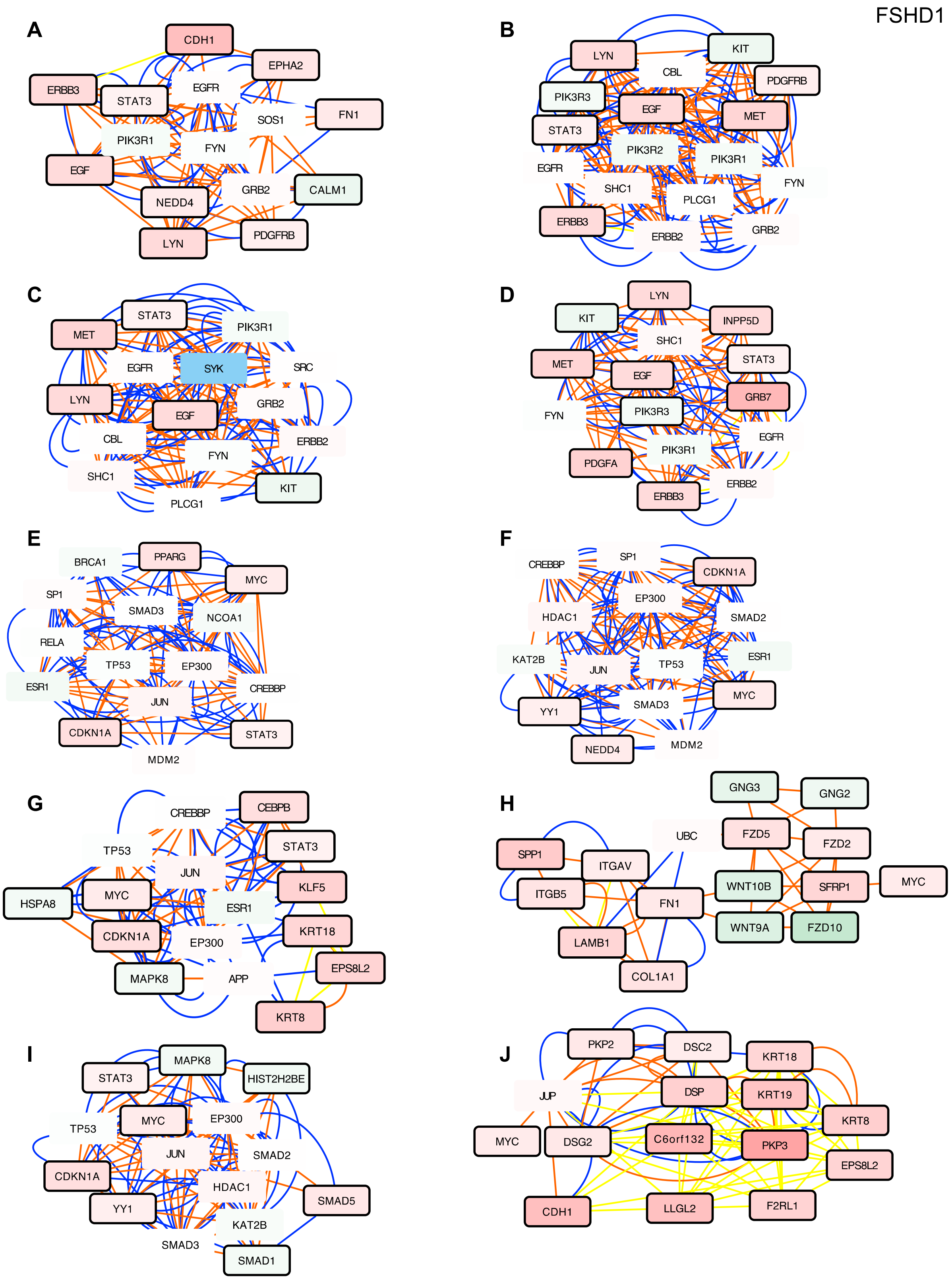

Supplementary figure S5. Active modules in FSHD1 cells. Representative active modules sampled from the accumulated Pareto front of 30 runs for the FSHD1 dataset using the MOGAMUN algorithm. The color of the edges (links) denotes the type of interaction/relationship between each pair of genes. Specifically, protein-protein interactions (blue links), biological pathways (orange links) and correlation of expression data (yellow links). Up-regulated nodes are colored in red, down-regulated ones, in green, the intensity of the color reflects the fold-change. Blue nodes represent the genes with no expression data available.

The black border reflects the level of significance (FDR<0.05 and -2>FC>2). **A-D.** Transmembrane receptor kinase signaling, **A**. p-value $1.85e^{-11}$. **B.** $6.02e^{-21}$. **C.** $3.29e^{-19}$. **D.** $1.14e^{-15}$. **E-F.** Response to exogenous stimuli. **E.** p-value $5.78e^{-13}$. **F.** $5.78e^{-13}$. **G.** Cellular response to organic substance, p-value $1.43e^{-8}$. **H.** Wnt signaling pathway, p-value $2.09e^{-7}$. **I.** Regulation of pri-miRNA transcription by RNA polII, p-value $2.82e^{-14}$. **J.** Cornification, p-value $3.044e^{-14}$.

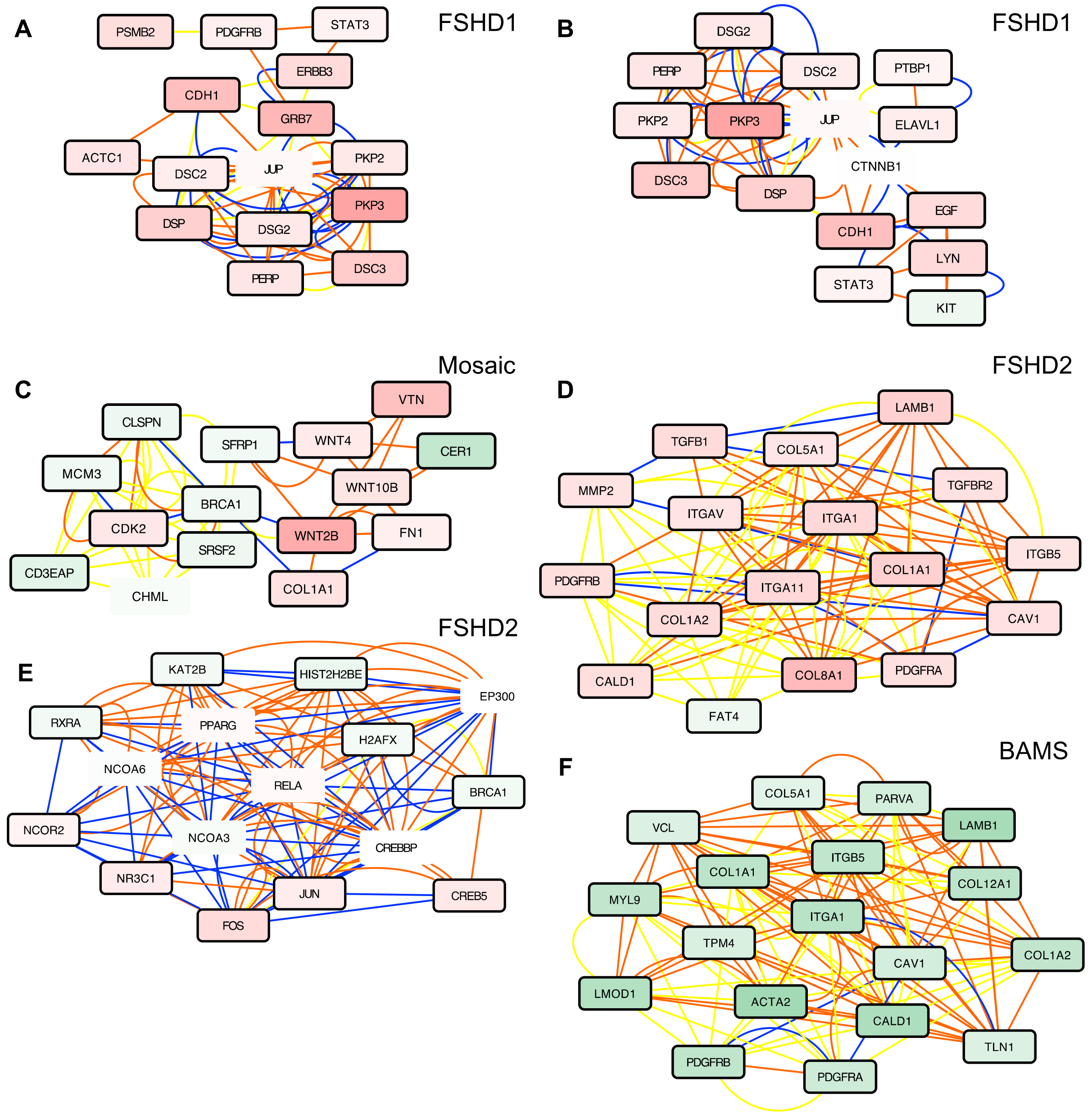

Supplementary figure S6. Additional active modules in FSHD1, FSHD1 mosaic, FSHD2 and BAMS NCSC. Representative active modules sampled from the accumulated Pareto front of 30 runs for the FSHD1, FSHD2, BAMS and mosaic datasets using the MOGAMUN algorithm. The color of the edges (links) denotes the type of interaction/relationship between each pair of genes. Specifically, protein-protein interactions (blue links), biological pathways (orange links) and correlation of expression data (yellow links). Up-regulated nodes are colored in red, down-regulated ones, in green, the intensity of the color reflects the fold-change. The black border reflects the level of significance (FDR<0.05 and -2>FC>2). **A-B.** Active modules of genes involved in desmosome organization**. A.** FSHD1, p-value $3.35e^{-14}$**. B.** FSHD1, p-value $5.2e^{-14}$. **C.** FSHD1 mosaic, Bone trabecula formation, p-value $8.5e^{-5}$. **D.** FSHD2, platelet derived growth factors**,** p-value $3.91e^{-11}$. **E.** FSHD2, positive regulation of transcription, p-value $3.8e^{-10.}$ **F.** BAMS, muscle contraction, p-value $6.33e^{-10}$.

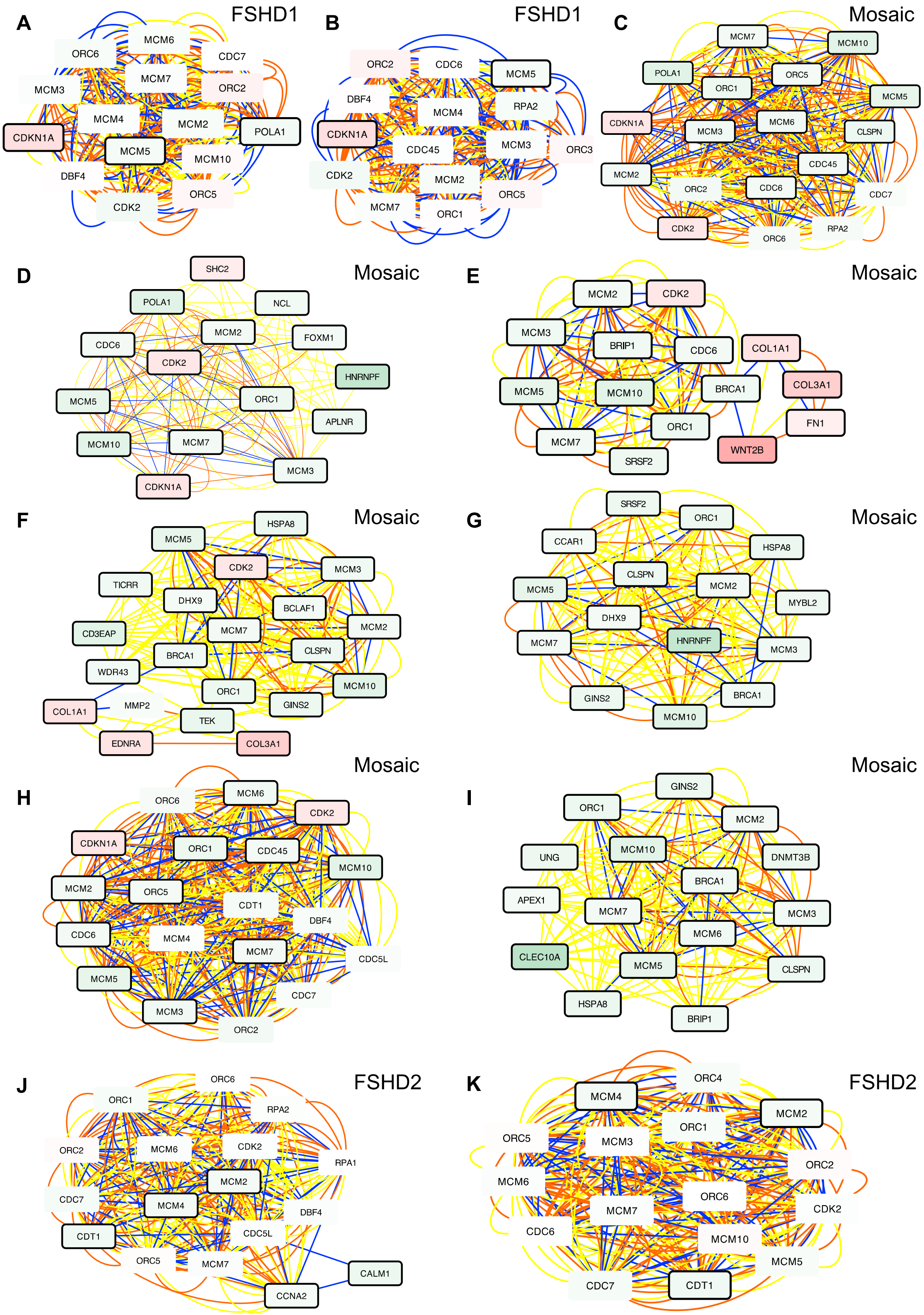

Supplementary figure S7. Additional active modules associated with replication in FSHD1, FSHD1 mosaic, FSHD2 and BAMS NCSC. Representative active modules sampled from the accumulated Pareto front of 30 runs for the FSHD1, FSHD2, and mosaic datasets using the MOGAMUN algorithm. The color of the edges (links) denotes the type of interaction/relationship between each pair of genes. Specifically, protein-protein interactions (blue links), biological pathways (orange links) and correlation of expression data (yellow links).

Up-regulated nodes are colored in red, down-regulated ones, in green, the intensity of the color reflects the fold-change. The black border reflects the level of significance (FDR<0.05 and -2>FC>2). **A-B.** FSHD1, p-value $4.3e^{-27}$**. C-I.** FSHD1 mosaic. **C.** p-value $7.7e^{-18}$. **D.** p-value $9.17e^{-32}$. **E.** p-value $5.08e^{-15}$. **F.** p-value $3.25e^{-14}$. **G.** p-value $3.3e^{-14}$. **H.** p-value $1.87e^{-34}$. **I.** p-value $7.1e^{-14}$. **J-K.** FSHD2. **J.** p-value $4.8e^{-15}$. **K.** p-value $6.8e^{-37}$.

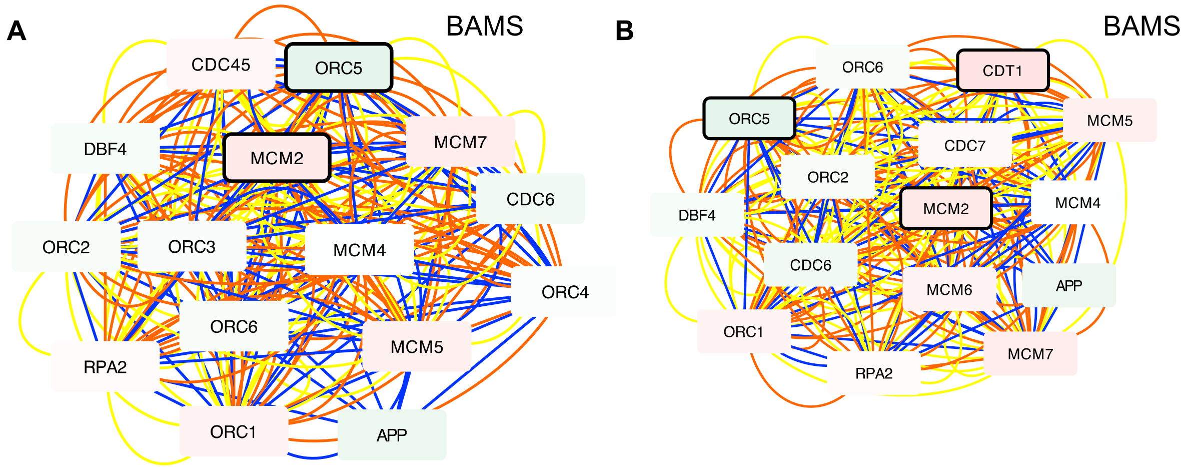

Supplementary figure S8. Additional active modules associated with replication in BAMS NCSCs. The color of the edges (links) denotes the type of interaction/relationship between each pair of genes. Specifically, protein-protein interactions (blue links), biological pathways (orange links) and correlation of expression data (yellow links). Up-regulated nodes are colored in red, down-regulated ones, in green, the intensity of the color reflects the fold-change. The black border reflects the level of significance (FDR<0.05 and -2>FC>2). **A.** p-value $4.32e^{-27}$**. B.** p-value $1.99e^{-25}$**.**
